# Extraperitoneal Single-Port Robot-Assisted Bilateral Retroperitoneal Lymph Node Dissection for Primary Non-Seminomatous Germ Cell Tumor: Technique and Surgical Considerations

**DOI:** 10.1590/S1677-5538.IBJU.2026.0242

**Published:** 2026-04-28

**Authors:** Lichen Chen, Kai Dong, Tongtong Zhang, Mingyue Tan, Dongliang Xu

**Affiliations:** 1 Shuguang Hospital Afiliated to Shanghai University of Traditional Chinese Medicine Urology Centre Shanghai China Urology Centre, Shuguang Hospital Afiliated to Shanghai University of Traditional Chinese Medicine, Shanghai, China; 2 Shuguang Hospital Affiliated to Shanghai University of Traditional Chinese Medicine Surgical Institute of Integrative Medicine Shanghai China Surgical Institute of Integrative Medicine, Shuguang Hospital Affiliated to Shanghai University of Traditional Chinese Medicine, Shanghai, China; 3 Shuguang Hospital Affiliated to Shanghai University of Traditional Chinese Medicine Surgical Institute Shanghai China Surgical Institute, Shuguang Hospital Affiliated to Shanghai University of Traditional Chinese Medicine, Shanghai, China; 4 Shanghai Key Laboratory of Traditional Chinese Clinical Medicine Shanghai China Shanghai Key Laboratory of Traditional Chinese Clinical Medicine, Shanghai, China

## Abstract

**Introduction:**

Retroperitoneal lymph node dissection (RPLND) is an established treatment option for clinical stage I–II non-seminomatous germ cell tumors (NSGCT), among which bilateral template dissection represents the most oncologically rigorous approach (1, 2). Robot-assisted RPLND has demonstrated reduced surgical morbidity while maintaining oncologic equivalence to open surgery (3). However, the prevailing multiport transperitoneal approach requires extensive bowel mobilization for bilateral exposure, adding operative complexity and bowel-related complication risk (4-6). A completely extraperitoneal single-port approach may circumvent these limitations by avoiding peritoneal entry and visceral manipulation (7).

**Materials and Methods:**

We present a 21-year-old male with clinical stage IIA NSGCT who underwent right radical orchiectomy and was found to have multiple enlarged retroperitoneal lymph nodes on CT.

The procedure was performed using the da Vinci Xi system with the patient in the supine position. A 4.0-cm right pararectal incision was made to access the retroperitoneal space, and a single-port multichannel device was deployed. Robotic instruments were introduced in a chopstick configuration, including a 30° endoscope, monopolar scissors, and bipolar forceps. The bilateral ureters and great vessels were first exposed to delineate the standard boundaries of the bilateral template while establishing an adequate retroperitoneal working space. A split-and-roll technique was then applied from the right ureter medially, progressively skeletonizing and preserving the vascular structures through sharp dissection to achieve complete lymphadenectomy within the bilateral template, with meticulous clipping of lymphatic channels.

**Results:**

Operative time was 175 minutes with an estimated blood loss of 35 mL. The patient resumed oral intake on postoperative day 1 and was discharged on day 2 (VAS: 2) without complications. Thirty-two lymph nodes were retrieved; three harbored teratoma (pN1). At 36-month follow-up, the patient remains disease-free with normal tumor markers and no retroperitoneal recurrence.

**Conclusions:**

To our knowledge, this represents the first reported extraperitoneal single-port bilateral robot-assisted RPLND. This approach achieves oncologically thorough dissection while minimizing morbidity and facilitating rapid recovery.

**Available at:**VIDEO

## Data Availability

Data Not Provided
